# Assessment of Public Stigma, Mental Health Literacy, and Help‐Seeking Intentions Based on Different Dimensions of Obsessive–Compulsive Content: A Study of the Spanish Mental Health‐Naïve Population

**DOI:** 10.1002/brb3.70159

**Published:** 2024-12-15

**Authors:** Antonio Chaves, Sandra Arnáez, Maria Roncero, Gemma García‐Soriano

**Affiliations:** ^1^ Departamento de Orientación Educativa IES Beatriu Civera, Conselleria d'Educació, Cultura i Esport Aldaia Valencia Spain; ^2^ Departamento de Personalidad, Evaluación y Tratamientos Psicológicos Universitat de València Valencia Spain

**Keywords:** content dimensions, help‐seeking, mental health literacy, OCD, public stigma, social distance

## Abstract

**Background:**

Obsessive–compulsive (OC) disorder is a debilitating disorder with a high delay in help‐seeking that could be associated with two barriers that may differ between OC content dimensions: public stigma and mental health literacy.

**Objectives:**

We aim to describe and analyze the differences among OC content dimensions in public stigma, social distance desire, mental health literacy, and help‐seeking intention in a sample of the mental health‐naïve population.

**Methods:**

A total of 487 participants from the Spanish community with no previous knowledge of or experience with OCD were randomly allocated one of six vignettes describing a person with OC symptoms of one out of six contents (i.e., aggression/harm; sexual; religious/blasphemous/immoral; contamination/washing; doubts/checking; or superstition/symmetry/order). After reading the vignette, participants completed the following questionnaires associated with their vignette: Attribution Questionnaire, General Help‐Seeking Questionnaire, Social Distance Scale, and Mental Health Literacy Questionnaire.

**Results:**

There were significant differences among OC content dimensions on the variables of interest. Aggression/harm OC content was associated with the highest public stigma and discriminating desire, and together with the religious/blasphemous/immoral OC contents were associated with the highest social distance desire. Although no differences appear between contents on intention to seek for formal treatment, participants would request less informal support for their sexual OC content than for the other categories. Contamination, doubts/checking, and superstition/symmetry/order OC contents were more frequently identified as OCD, although only around 50% or less of the sample identified the sexual, superstition/symmetry/order, doubts/checking, and religious/blasphemous/immoral OC contents as a mental health problem.

**Conclusions:**

Interventions to reduce stigma and increase help seeking are necessary and should consider OC heterogeneity.

## Graphical abstract

Stigma and content heterogeneity in OCD.

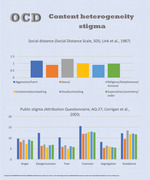

## 1 Introduction

Obsessive–compulsive disorder (OCD) is characterized by the presence of obsessions, which are repetitive unwanted intrusive thoughts, images or urges, and/or compulsions, defined as ritualized behaviors intended to reduce distress (American Psychiatric Association 2013). OCD has a heterogeneous symptom presentation that could be grouped into a two‐factor solution: autogenous obsessions containing aggressive, sexual, religious/blasphemous/immoral themes, and reactive contents including contamination/washing, doubts and checking, need for symmetry/ordering, and superstition content dimensions (Belloch, Morillo, and García‐Soriano 2007; Garcia‐Soriano and Belloch 2013; García‐Soriano and Belloch 2013). It affects approximately 2%–2.3% of the population (Ruscio et al. 2010) and although it is reported as one of the 10 leading causes of disability worldwide (World Health Organization 1999), between 38% and 89.8% of adults with OCD do not ask for treatment for their symptoms (García‐Soriano et al. 2014; Subramaniam et al. 2012) and take between 3.29 (Spain) and 17 (USA) years to seek treatment or receive adequate treatment (García‐Soriano et al. 2014).

Treatment‐seeking behavior might differ depending on the OC content dimensions experienced by patients. In this sense, different studies suggest that patients seeking treatment showed a higher frequency of aggression/harm and religious obsessions (Beşiroğlu, Çilli, and Aşkin 2004) and that help‐seeking is predicted by severe obsessions of violence (Mayerovitch et al. 2003); although other studies do not find these associations (Levy et al. 2013). Untreated OCD is associated with significant functional impairment and reduction in quality of life (Hollander et al. 1996; Macy et al. 2013), with a low rate of spontaneous remission (Melkonian et al. 2022). The delay in help‐seeking behavior in patients with OCD is explained by different variables associated with the stigma and poor mental health literacy associated with OCD (Belloch et al. 2009; García‐Soriano et al. 2014; Williams and Turkheimer 2007).

Stigma has been defined as “the prejudice and discrimination that robs people with mental illness of opportunities to accomplish personal goals” (Corrigan et al. 2015, 635). Stigma comprise at least four components, such as self‐stigma/internalized stigma, stigma by association, structural stigma (systemic or institutional), and public/interpersonal stigma (Thornicroft et al. 2022). Public stigma occurs when the general population agrees with negative stereotypes and maintains negative reactions toward people with mental disorders. This consists of stereotypes, such as perceiving a person with a mental disorder as being dangerous; prejudices or affective reactions, such as feeling scared of someone with a mental disorder; and discriminating behaviors, such as segregating or maintaining social distance from these individuals (Link et al. 1987). Studies in this field have used descriptions of OCD symptom dimensions (e.g., aggression/harm or contamination/washing contents) and asked questions (mainly through different versions of the Attribution Questionnaire or Social Distance Scale) about the described vignettes (e.g., Coles et al. 2023; Durna, Yorulmaz, and Aktaç 2019). While some studies have been carried out on the general population (McCarty et al. 2017) or undergraduate students (García‐Soriano and Roncero 2017), others have used specific sample groups such as clinicians (Steinberg and Wetterneck 2017). A recent review indicates that aggression/harm and sexual OC content dimensions are associated with a higher desire for social distance, whereas aggression/harm is associated with greater perceived dangerousness than symmetry/just right (Ponzini and Steinman 2022). These findings were further supported by two recent studies that explored stigma in a wider range of obsessional content than previous studies. In this sense, it was found that sexual and harm/aggression OC contents were associated with serious mental‐illness stigma, whereas religious/scrupulous contents were associated with anxiety‐relevant stigma (Ponzini et al. 2024). Furthermore, unacceptable OC contents were more stigmatized than contamination contents (Coles et al. 2023). Thus, it could be argued that at least some autogenous contents are associated with higher levels of public stigma and social distance than reactive contents (Ponzini and Steinman 2022).

Furthermore, mental health literacy, defined as “knowledge and beliefs about mental disorders, aiding their recognition, management or prevention” (Jorm et al. 1997, 182), also differs among OC content dimensions. In this sense, it has been suggested that contamination, order, or checking OCD dimensions are better recognized than autogenous ones (Coles et al. 2023; García‐Soriano and Roncero 2017; McCarty et al. 2017). Even mental health professionals tend to better identify contamination in comparison with sexual, aggression, and religious contents, which are more frequently misidentified (Glazier et al. 2013).

Although these studies suggest that autogenous contents are more associated with stigmatizing attitudes and lower recognition in the general population, little is known about specific stigmatizing dimensions or literacy variables. It is also difficult to reach further conclusions regarding OCD content heterogeneity, as studies use a variety of methods and only a few include more than three or four different OC contents (Coles et al. 2023; McCarty et al. 2017; Ponzini et al. 2024). Furthermore, most of these studies have been carried out on North American samples, and none of them explored any intention to seek treatment in the event of experiencing OC symptoms.

Given the above‐mentioned limitations, it is necessary to provide better knowledge of the variables associated with delay in seeking help as well as information about the intention to seek help in a Spanish community sample. We are interested in exploring these variables in a sample of individuals with no previous experience with or knowledge about OCD, as prior research suggests that individuals with experience or contact with mental health services/providers (e.g., Arnáez et al. 2023; García‐Soriano and Roncero 2017) or those with knowledge about mental health, often exhibit fewer stigmatizing attitudes (Arnáez et al. 2023). In fact, psychoeducation is a frequently used variable to combat stigma (Griffiths et al. 2014). In this vein, this study aims to describe and analyze the levels of public stigma, social distance desire, mental health literacy, and help‐seeking intention associated with OCD, taking into consideration the OC content dimensions in people who are mental‐health naïve.

We hypothesize differences in the variables of interest depending on the OC content dimension. In this sense, and based on previous results in the literature, public stigma and social distance will be higher in relation to aggression/harm OC content, whereas mental health literacy will be higher for contamination/washing OC content (Coles et al. 2023; Ponzini et al. 2024). Furthermore, as some studies suggest that aggression/harm OC contents are associated with a higher rate of seeking help (Beşiroğlu, Çilli, and Aşkin 2004), we assume that the community will show a higher tendency to seek help when experiencing obsessions related to aggression/harm. Whether or not they identify their thoughts as obsessions, they may be motivated to seek help, either because they are afraid of being dangerous, or because they perceive their symptoms as more distressing and unacceptable.

## 2 Materials and Methods

### 2.1 Sample and Recruitment

Data were collected from the Spanish general population through social networks and media channels as part of a study that aimed to analyze the efficacy of a mobile application named esTOCma v.1.0 (Chaves et al. 2022; Chaves, Arnáez, and García‐Soriano 2024; García‐Soriano et al. 2024). This application was designed as a serious game to combat the stigma associated with OCD and to increase awareness of the disorder.

Participants had to download the mobile application and sign the informed consent. Participants that met the inclusion criteria of being over 18 years old and self‐reporting as neither having an OCD diagnosis nor having suspected symptoms of OCD were randomized by the app using a sampling without replacement method to one of six standardized vignettes describing a person with OC symptoms from one out of six contents (i.e., aggression/harm [aggression toward his/her partner and him/herself], sexual [images of unpleasant sexual content with unwanted persons including having oral sex with his/her father], religious/blasphemous/immoral [insulting God and cursing other people], contamination/washing [fear of becoming contaminated and transmitting contamination to other people], doubts/checking [doubts about gas, taps or doors], or superstition/symmetry/order [need for things to be arranged in a certain way for fear of something bad happening to his/her family]) based on real clinical descriptions of OCD patients (Belloch, Cabedo, and Carrió 2011). All patients described in the vignettes were named A, their gender was not specified (as in Spanish it is possible to use impersonal sentences since pronouns may be omitted or do not change depending on whether the subject is male or female), all were the same age (i.e., A is middle‐aged), and all described symptoms that met the diagnostic criteria for OCD according to DSM‐5, although the labels OCD, obsessions, and compulsions were avoided. All patients described a similar severity of symptomatology, with similar interference with social, family, and work domains; associated distress; and time consumed by obsessions and compulsions. Furthermore, all vignettes adhere to a uniform structure. Initially, a description of the obsessions and compulsions is provided, followed by an explanation of the interference and impairment of quality of life. In addition, all the vignettes are of a similar length in Spanish, between 166 and 175 words. The translation of the vignettes into English has been included in the Supporting Information. Participants then completed the sociodemographic and assessment instruments, most of which were with reference to the assigned vignette. A total of 933 participants (*M*_age_ = 37; SD = 16.61; 66.6% women) met the inclusion criteria and completed the assessment protocol. The following exclusion criteria were then applied: having knowledge regarding OCD, inferred from the fact that the person self‐reported any kind of mental health training (*n* = 255), and/or being a relative/friend of a person with an OCD diagnosis (*n* = 250). Exclusion criteria were defined based on previous research that shows that people with previous knowledge or experience/contact with mental health services/providers show less stigmatizing attitudes (Arnáez et al. 2023; García‐Soriano and Roncero 2017).

The final study sample consisted of 487 participants (*M*_age_ = 33.84; SD = 16.34; 62.8% women; 74.7% high education level) with no previous knowledge of mental health or contact with OCD (see Table 1). These participants were distributed in the vignettes in the following way: aggression/harm, *n* = 83; sexual, *n* = 84; religious/blasphemous/immoral, *n* = 76; contamination/washing, *n* = 81; doubts/checking, *n* = 91; or superstition/symmetry/order, *n* = 72. Demographic data are displayed in Table 1.

**TABLE 1:** Demographic characteristics by assigned vignette.

	Vignette content						
	Total (*N* = 487)	Aggression/harm (*n* = 83)	Sexual (*n* = 84)	Religious/blasphemous/immoral (*n* = 76)	Contamination/washing (*n* = 81)	Doubts/checking (*n* = 91)	Superstition/symmetry/order (*n* = 72)
	Mean [SD] or *n* (%)	Mean [SD] or *n* (%)	Mean [SD] or *n* (%)	Mean [SD] or *n* (%)	Mean [SD] or *n* (%)	Mean [SD] or *n* (%)	Mean [SD] or *n* (%)
Age (years)	33.84 [16.34]	32.17 [15.96]	35.00 [16.25]	33.29 [17.52]	35.20 [17.00]	32.67 [15.12]	34.92 [16.60]
Gender							
Men	174 (35.7)	27 (32.5)	29 (34.5)	26 (34.2)	27 (33.3)	39 (43.8)	26 (36.1)
Women	306 (62.8)	56 (67.5)	55 (65.5)	47 (61.8)	54 (66.7)	50 (56.2)	44 (61.1)
Other	2 (0.4)	—	—	—	—	2 (2.2)	—
Prefer not to say	5 (1.0)	—	—	3 (3.9)	—	—	2 (2.8)
Education level							
Primary school	(41) 8.4	5 (6)	9 (10.7)	3 (3.9)	5 (6.2)	9 (9.9)	10 (13.9)
High school	(82) 16.8	16 (14.5)	10 (11.9)	16 (21.1)	13 (16.0)	19 (20.9)	12 (16.7)
University level	(364) 74.7	66 (79.5)	65 (77.4)	57 (75.0)	63 (77.8)	63 (69.2)	50 (69.4)

This study was approved by the Ethics Committee of Research in Humans of the Ethics Commission in Experimental Research of the Universitat de València.

### 2.2 Measures

*Attribution Questionnaire* (AQ‐27) (Corrigan et al. 2003). This measures public stigma associated with the vignette. The scale includes 27 items rated on a 9‐point Likert scale from 1 to 9. It includes nine subscales (scores ranging from 3 to 27): *Responsibility*, *Pity*, *Anger*, *Dangerousness*, *Fear*, *No help*, *Coercion*, *Segregation*, and *Avoidance*. Higher scores indicate higher public stigma. The Spanish validation (Muñoz et al. 2015) has shown good psychometric properties. In this study, most of the subscales demonstrated acceptable to good internal consistency (Cronbach's *α* ranging from 0.68 [*Avoidance*] to 0.89 [*Fear*]). However, *Responsibility* (Cronbach's *α* = 0.49) and *Pity* (Cronbach's *α* = 0.39) exhibited a poor internal consistency. Due to their potential unreliability, these two subscales will not be included in the subsequent analysis. The internal consistency of the total scale (excluding *Responsibility* and *Pity* items) was excellent, with a Cronbach's *α* of 0.90.

*Social Distance Scale* (SDS) (Link et al. 1987). This assesses an individual's willingness to interact with the individual described in the vignette across seven different situations on a Likert Scale from 0 (*definitely willing*) to 3 (*definitely unwilling*). A total score has been calculated as a mean (ranging from 0 to 3) with higher scores indicating greater preference for social distance. In the current study, the SDS showed adequate internal consistency (Cronbach's *α* = 0.87).

*General Help‐Seeking Questionnaire* (GHSQ) (Wilson and Deane 2005). This assesses the intention to seek help from 10 different formal and informal sources. Formal sources include mental health professionals (psychologists or psychiatrists) or doctors/general practitioners, while informal sources include, for example, friends or parents. Participants rate their likelihood of seeking help regarding a vignette from 1 (*extremely unlikely*) to 7 (*extremely likely*). Higher scores indicate a higher intention to seek help. It includes a total score calculated as a mean score and two subscales: formal sources and informal sources of help (scores ranging from 1 to 7). The Spanish translation has been used (Pacheco del Castillo 2017). In the present study, the GHSQ showed adequate internal consistency for the total score (Cronbach's *α* = 0.76).

*Mental Health Literacy Questionnaire* (MHL Questionnaire) (Chaves et al. 2022). This is an instrument developed ad hoc based on previous studies (Chaves et al. 2021; García‐Soriano and Roncero 2017) and assesses one of the components defined by Kutcher, Bagnell, and Wei (2015) as MHL but only referring to OCD: the understanding of OCD and its treatment. It is made up of two parts. Part 1 has four multiple‐choice questions, each with between two and seven alternative answers, only one of which is correct. The questions refer to the assigned vignette (Person A) and evaluate the following dimensions: (1) problem recognition (Is *what happens to A: a cause for concern?* [Response alternatives: yes, no, I do not know]); (2) OCD identification (*What do you think might be happening to A?* [Response alternatives: family problem, adjustment problem, anxiety disorder, generalized anxiety disorder, schizophrenia, obsessive–compulsive disorder, depression]); (3) perception of causality (*What do you think is the primary cause that triggers A's problem?* [Response alternatives: stress, having experienced trauma, her/his personality, family problems, an organic/physical problem, a mental health problem)]; and (4) effective treatment option (*In your opinion, which treatment would be most helpful for A?* [Response alternatives: medication/drugs, leisure activities, sport, relaxation, meditation, or stress management courses, psychological treatment]). Part 2 consists of four multiple‐choice questions with three alternative answers, of which only one is correct, and pertains to participants' general knowledge of OCD, that is, it does not refer to the vignette: (1) identification of OCD as a mental disorder (*Obsessive–Compulsive Disorder is*: (a) A learning disorder, (b) A mental disorder, (c) A set of manias); (2) definition of an obsession (*Obsessions are*: (a) Unwanted daily life preoccupations, (b) Hallucinations, (c) Unwanted repetitive thoughts, images or urges); (3) definition of a compulsion (*Compulsions are*: (a) Repetitive behaviors with the main goal of attracting attention, (b) Repetitive behaviors to reduce the discomfort of the obsessions, (c) Repetitive behaviors of no importance); and (4) the role played by compulsions and other control strategies in the maintenance of obsessions (*The strategies and compulsions that the person carries out in order to feel better achieve in the long term*: (a) OCD becomes stronger, (b) That the person does not have anxiety, (c) That the person does not have obsessions). Part 2 was not used in this study as it was not answered based on the vignettes and comparisons between OCD contents were not possible.

### 2.3 Statistical Analysis

Analysis was performed with SPSS version 28. Descriptive statistics were used to report the means and standard deviations of the main variable measures. Analyses of variance were conducted for quantitative variables, and chi‐square tests (*χ* ^2^) were used for qualitative variables (i.e., MHL analyses at item level) to explore differences in vignette obsessive contents. The post hoc Bonferroni test was used to carry out multiple comparisons between vignettes. Effects sizes were calculated using partial *η* ^2^ values. To assess the statistical significance of our findings, we employed a pre‐defined significance level of 0.05 (*α* level).

## 3 Results

### 3.1 Levels of Public Stigma, Social Distance, and Help‐Seeking Associated With Different Dimensions of Obsessive Contents

Regarding public stigma associated with OCD, as measured by the AQ‐27, the coercion subscale scored the highest and the segregation subscale the lowest (Table 2). Statistically significant differences across OC content dimensions were observed in the AQ‐ 27 total score (*F*_[5, 481]_ = 8.79, *p* < 0.001, $\eta_{p}^{2}=0.08$) and in the all subscales: anger (*F*_[5, 481]_ = 3.03, *p* = 0.011, $\eta_{p}^{2}=0.03$), dangerousness (*F*_[5, 481]_ = 26.95, *p* < 0.001, $\eta_{p}^{2}=0.22$), fear (*F*_[5, 481]_ = 16.44, *p* < 0.001, $\eta_{p}^{2}=0.15$), intention not to help (*F*_[5, 481]_ = 2.23, *p* = 0.050, $\eta_{p}^{2}=0.02$), coercion (*F*_[5, 481]_ = 4.48, *p* < 0.001, $\eta_{p}^{2}=0.05$), segregation (*F*_[5, 481]_ = 4.80, *p* < 0.001, $\eta_{p}^{2}=0.05$), and avoidance (*F*_[5, 481]_ = 4.81, *p* < 0.001, $\eta_{p}^{2}=0.05$).

**TABLE 2:** Means and standard deviations of the vignette obsessive contents (*n* = 487).

Variable	Vignette content						
	Total (*N* = 487)	Aggression/harm (*n *= 83)	Sexual (*n *= 84)	Religious/blasphemous/immoral (*n *= 76)	Contamination/washing (*n *= 81)	Doubts/checking (*n *= 91)	Superstition/symmetry/order (*n *= 72)
AQ‐27							
Anger	8.75 (4.65)	9.78 (4.72)	8.21 (4.46)	9.15 (4.31)	7.30 (4.29)	9.30 (4.85)	8.73 (4.95)
Dangerousness	7.53 (4.82)	12.40 (5.17)	6.38 (3.97)	7.01 (4.52)	5.67 (3.37)	6.73 (4.05)	6.88 (4.40)
Fear	6.69 (4.54)	10.34 (5.55)	5.96 (4.19)	6.72 (4.08)	4.96 (2.78)	6.21 (4.16)	5.87 (4.01)
No help	8.30 (4.27)	7.37 (3.60)	8.33 (4.32)	9.48 (4.68)	8.03 (3.91)	8.05 (4.56)	8.70 (4.27)
Coercion	13.05 (5.75)	15.61 (5.80)	11.97 (5.89)	12.18 (5.61)	12.80 (4.86)	13.00 (6.01)	12.65 (5.54)
Segregation	6.25 (4.33)	8.21 (5.27)	5.48 (4.08)	6.47 (4.55)	6.00 (4.02)	5.74 (3.62)	5.58 (3.71)
Avoidance	11.84 (5.39)	12.25 (5.42)	9.60 (4.99)	13.59 (5.15)	11.81 (5.37)	12.14 (5.44)	11.79 (5.33)
Total	62.44 (23.45)	76.00 (26.57)	55.96 (22.23)	64.63 (22.14)	56.60 (20.35)	61.20 (21.36)	60.23 (22.21)
SDS							
Total score	1.04 (0.64)	1.19 (0.66)	0.90 (0.63)	1.32 (0.63)	1.00 (0.59)	0.90 (0.57)	0.98 (0.69)
GHSQ							
Formal sources	5.56 (1.34)	5.55 (1.45)	5.48 (1.28)	5.40 (1.46)	5.58 (1.39)	5.76 (1.11)	5.55 (1.38)
Informal sources	4.57 (1.45)	4.86 (1.28)	3.92 (1.62)	4.60 (1.49)	4.63 (1.35)	4.79 (1.44)	4.65 (1.30)
Total score	4.29 (1.03)	4.40 (1.05)	4.03 (1.09)	4.28 (1.08)	4.22 (0.97)	4.45 (0.95)	4.32 (1.01)

*Note*: Data as mean and standard deviation (SD).

Abbreviations: AQ‐27 = Attribution Questionnaire, GHSQ = General Help Seeking Questionnaire, SDS = Social Distance Scale.

Post‐hoc comparisons showed that regarding the AQ‐27 total score, the aggression/harm content (versus all the other content dimensions) scored the highest (ranging from *p* = 0.024 to *p* < 0.001). When AQ‐27 subscales were examined, the aggression/harm (vs. other content dimensions) was the only subscale that showed significantly higher scores in all stigma subscales (ranging from *p* = 0.024 to *p* < 0.001). Specifically, aggression/harm was associated with higher scores than all the other obsessive contents on dangerousness (all *p* < 0.001), fear (all *p* < 0.001), coercion (ranging from *p* = 0.036 to *p* < 0.001), and segregation (ranging from *p* = 0.014 to *p *< 0.001; although did not differ from moral, *p* = 0.151); higher avoidance than sexual contents (*p* = 0.20); higher anger than contamination/washing (*p* = 0.010); and higher intention not to help than religious/blasphemous/immoral (*p* = 0.027). Furthermore, sexual contents were associated with lower avoidance than religious/blasphemous/immoral contents (*p* < 0.001) and doubts/checking contents (*p* = 0.25).

The desired social distance, measured with the SDS, showed statistically significant differences across OC content dimensions (*F*_[5, 481]_ = 5.89, *p* < 0.001, $\eta_{p}^{2}=0.06$). Post hoc tests revealed that the aggression/harm vignette was associated with higher social distance desire than the sexual (*p* = 0.044) and doubts/checking (*p* = 0.036) contents. Moreover, the religious/blasphemous/immoral vignette was associated with a higher social distance desire than the other OC content dimensions (ranging from *p* = 0.022 to *p* < 0.001), except for aggression/harm content (*p* = 0.999).

In terms of help‐seeking intention (GHSQ), no significant differences were observed across OC content dimensions in the total score (*F*_[5, 481]_ = 1.80, *p* = 0.110, $\eta_{p}^{2}=0.02$) or in seeking help from formal sources (*F*_[5, 481]_ = 0.70, *p* = 0.617, $\eta_{p}^{2}=0.01$). However, significant differences were found in terms of informal sources (*F*_[5, 481]_ = 4.67, *p* < 0.001, $\eta_{p}^{2}=0.05$). Specifically, post hoc analysis revealed a lower tendency to seek help from informal sources when experiencing sexual OC content compared to other dimensions (ranging from *p* = 0.022 to *p* < 0.001).

### 3.2 Mental Health Literacy Associated With Different Dimensions of Obsessive Contents

Regarding mental health literacy (Table 3), the results showed that after reading the vignette describing a person with OC symptoms, most of the participants were able to recognize that a problem existed, with no statistically significant differences among the different OC content vignettes. Regarding the identification of OCD, a quarter of the participants did not correctly identify the vignette as describing a person with OCD. Also, the aggression/harm and sexual vignettes were the least frequently identified as OCD. As for the cause of the problem described in the vignette, over half of the participants identified a mental health problem. Significant differences between the OC content vignettes appeared, and again, the sexual contents were the least frequently identified as a mental health problem. Between 29.6% (contamination/washing vignette) and 60.7% (sexual vignette) of participants failed to identify the problem as a mental health problem. Finally, close to 80% of the participants selected psychological treatment as the most effective treatment. Significant differences were found between OC content vignettes. About 20% of participants in the vignettes describing doubts/checking and superstition/symmetry/order contents identified relaxation/meditation as the preferred treatment, with misidentification rates ranging between 22% and 27.8%.

**TABLE 3:** Percentages of the responses by item to the Mental Health Literacy (MHL) Questionnaire Part 1 for the whole sample, depending on the vignette content.

	Total (*N* = 487)	Aggression/harm (*n *= 83)	Sexual (*n *= 84)	Religious/blasphemous/immoral (*n *= 76)	Contamination/washing (*n *= 81)	Doubts/checking (*n *= 91)	Superstition/symmetry/order (*n *= 72)	*χ* ^2^_(487)_	*p*
Problem recognition (MHL Part 1—Item 1)								*χ* ^2^ (10) = 12.90	0.229
**Recognition as a problem**	81.1 (395)	80.7 (67)	84.5 (71)	81.6 (62)	81.5 (66)	79.1 (72)	79.2 (57)		
No recognition as a problem	6 (29)	2.4 (2)	1.2 (1)	7.9 (6)	8.6 (7)	5.5 (5)	11.1 (8)		
I do not know	12.9 (63)	16.9 (14)	14.3 (12)	10.5 (8)	9.9 (8)	15.4 (14)	9.7 (7)		
*Misidentification rate*	*18.9 (92)*	*19.3 (16)*	*15.5 (13)*	*18.4 (14)*	*18.5 (15)*	*20.9 (19)*	*10.8 (15)*		
OCD identification (MHL Part 1—Item 2)								*χ* ^2^ (30) = 145.05	≤ 0.001
Family problem	2.3 (11)	0 (0)	11.9 (10)	0 (0)	0 (0)	1.1 (1)	0 (0)		
Adjustment problem	3.7 (18)	2.4 (2)	6.00 (5)	3.9 (3)	1.2 (1)	2.2 (2)	6.9 (5)		
Anxiety disorder	7.2 (35)	10.8 (9)	4.8 (4)	6.6 (5)	8.6 (7)	6.6 (6)	5.6 (4)		
Generalized Anxiety Disorder	4.3 (21)	8.4 (7)	7.1 (6)	5.3 (4)	1.2 (1)	2.2 (2)	1.4 (1)		
Schizophrenia	5.7 (28)	25.3 (21)	1.2 (1)	5.3 (1)	1.2 (1)	1.1 (1)	0 (0)		
**Obsessive–compulsive disorder**	74.5 (363)	50.6 (42)	66.7 (56)	73.7 (56)	87.7 (71)	85.7 (78)	83.3 (60)		
Depression	2.3 (11)	2.4 (2)	2.4 (2)	5.3 (4)	0 (0)	1.1 (1)	2.8 (2)		
*Misidentification rate*	*25.5 (124)*	*49.4 (41)*	*33.3 (28)*	*26.3 (20)*	*12.3 (10)*	*14.4 (13)*	*16.7 (12)*		
Perception of causality (MHL Part 1—Item 3)								*χ* ^2^ (25) = 79.29	≤ 0.001
Stress	10.1 (49)	12 (10)	6 (5)	5.3 (4)	12.3 (10)	13.2 (12)	11.1 (8)		
Trauma	22.2 (108)	10.8 (9)	47.6 (40)	15.8 (12)	11.1 (9)	23.1 (21)	23.6 (17)		
Personality	8.6 (42)	3.6 (3)	3.6 (3)	14.5 (11)	6.2 (5)	9.9 (9)	15.3 (11)		
Family problems	1 (5)	0 (0)	2.4 (2)	3.9 (3)	0 (0)	0 (0)	0 (0)		
Organic alteration	1.8 (9)	3.6 (3)	1.2 (1)	1.3 (1)	0 (0)	3.3 (3)	1.4 (1)		
**Mental health problem**	56.3 (274)	69.9 (58)	39.3 (33)	59.2 (45)	70.4 (57)	50.5 (46)	48.6 (35)		
*Misidentification rate*	*43.7 (213)*	*30.1 (25)*	*60.7 (51)*	*40.8 (31)*	*29.6 (24)*	*49.5 (45)*	*51.4 (37)*		
Effective treatment (MHL Part 1—Item 4)								*χ* ^2^ (20) = 43.86	0.002
**Medication**	3.1 (15)	10.8 (9)	1.2 (1)	1.3 (1)	1.2 (1)	2.2 (2)	1.4 (1)		
Leisure activities	1.8 (9)	2.4 (2)	1.2 (1)	2.6 (2)	0 (0)	0 (0)	5.6 (4)		
Sport	2.7 (13)	4.8 (4)	0 (0)	2.6 (2)	1.2 (1)	3.3 (3)	4.2 (3)		
Relaxation/meditation	13.3 (65)	12 (10)	8.3 (7)	7.9 (6)	14.8 (12)	18.7 (17)	18.1 (13)		
**Psychological treatment**	79.1 (385)	69.9 (58)	89.3 (75)	85.5 (65)	82.7 (67)	75.8 (69)	70.8 (51)		
*Misidentification rate*	*17.8 (87)*	*19.3 (16)*	*9.5 (8)*	*11.9 (10)*	*16.1 (13)*	*22 (20)*	*27.8 (20)*		

*Note*: Data as % (*n*). In bold correct alternative. Misidentification rates (in *italics*) were calculated summing up the alternatives not in bold, that is the incorrect answers.

Abbreviation: MHL Part 1 = Part 1 of the Mental Health Literacy Questionnaire.

## 4 Discussion

The present study aims to describe and analyze the levels of public stigma, social distance desire, mental health literacy, and help‐seeking intention associated with OCD, taking into consideration six different OC content dimensions in a Spanish sample of people who were mental‐health naïve. We aim to provide a better understanding of the variables associated with delays in seeking help, as well as to offer information on help‐seeking intentions. This study extends previous research comparing a higher number of OC dimensions in three relevant variables, including help‐seeking intentions, a variable not previously explored, in an OCD naïve sample outside America.

The results suggest a tendency to experience stereotypes and prejudices toward a person experiencing OCD. This therefore suggests the need to develop programs to fight stigma, especially addressing the dimensions with the highest stigmatizing scores: the intention to force the person to initiate treatment and to avoid people with OCD. Exploring differences in stigma among OC content dimensions will help us to better define the characteristics that these programs should have. As hypothesized, stigma was higher in relation to the aggression/harm content dimension than all other OC dimensions. Specifically, participants appraised the person showing aggression/harm symptoms as more dangerous and, probably as a consequence, experienced higher fear and anger, with a higher intention to use discriminating behaviors such as coercion, avoidance, or segregation from the community. These results extend previous studies reporting higher levels of fear and dangerousness associated with aggression/harm contents as opposed to specific contents such as contamination (McCarty et al. 2017), religious (Coles et al. 2023), or symmetry/order OC contents (García‐Soriano and Roncero 2017; Homonoff and Sciutto 2019). In a study using a total stigma score (AQ‐9), Ponzini et al. (2023) reported higher stigmatizing attitudes associated with aggressive and sexual contents than contamination and symmetry OC contents. Our data do not support the idea that aggressive and sexual content present similar stigmatizing attitudes, perhaps due to differences in the variables analyzed in the AQ‐27 at the subscale level versus the AQ‐9 at the global score, or differences in the content of the sexual vignette. In our case, it was associated with images of a person having oral sex with his/her own father while that of Ponzini et al. (2023) described a male who had thoughts about impulsively having sex with his children. It could be that in this case, as in the aggressive vignettes, although the egodystonicity of the thoughts is clear, participants may be implying that the person could lose control and act on his/her obsessions (described in the vignette as unwanted thoughts and images), thus being dangerous to him/herself, his/her children, or the community. Despite these differences, which future studies should address by exploring the heterogeneity of sexual OC contents, these results imply that future interventions need to take into account the heterogeneity of OCD symptoms, and specifically address OCD with aggression/harm contents.

In our study, there was a higher intention to maintain social distance with people showing aggression/harm and religious/blasphemous/immoral OC content dimensions than with the other OC dimensions. Our results are partially consistent with Durna, Yorulmaz, and Aktaç (2019), who have reported that aggression/harm and sexual contents (in both cases related to aggression toward children) were associated with higher social distance intention than other symptom dimensions such as doubts/checking and contamination/washing. A previous study also found a higher social distance desire associated with sexual OC contents related to strangers than contamination/washing, doubts/checking, or symmetry/incompleteness (McCarty et al. 2017). However, in our study, social distance associated with sexual OC contents was similar to that associated with doubts/checking, and lower than that associated with aggression/harm or religious/blasphemous/immoral contents. Again, results suggest not only the need to especially work on aggression/harm but also on religious/blasphemous/immoral contents when developing programs to fight against stigma.

Furthermore, results emphasize the need to publicize and facilitate the comprehension of empirically validated treatments for OCD and their effectiveness, as participants reported that they were not sure whether they would ask for help if experiencing OCD. This result is consistent with data suggesting that a high percentage of patients do not ask for help (Mayerovitch et al. 2003). Interestingly, it seems that participants were more likely to ask for formal than informal sources of help, consistent with studies analyzing disorders different from OCD (Gorczynski, Sims‐Schouten, and Wilson 2020). Results suggest that the intention to seek help does not differ among different OC contents, not confirming our hypothesis. However, they align with studies conducted on clinical samples and that did not find an association between help‐seeking and OC contents (Levy et al. 2013). Differences in the intention to seek help only appeared regarding a lower tendency to seek help from informal sources in the sexual contents, consistent with this dimension being associated with guilt and shame (Simonds and Thorpe 2003).

In terms of mental health literacy, results show that around three quarters of participants identified the vignette as a problem that needs attention, recognizing that it represents OCD, and would recommend psychological treatment; although only about 50% recognized that it represents a mental health problem. Previous studies on community samples have reported similar results (Coles, Heimberg, and Weiss 2013). Regarding the specific contents, as hypothesized and consistent with previous studies (Coles et al. 2023; McCarty et al. 2017), contamination/washing was the content most frequently identified as a mental disorder and as OCD. These data could suggest that participants might be more familiar with contamination symptoms, probably due to the influence of the media, which often portrays OCD primarily through the contamination dimension (Cathey and Wetterneck 2013; Fennell and Boyd 2014). Regarding doubts/checking and superstition/symmetry dimensions, although frequently identified as OCD (García‐Soriano and Roncero 2017), only about half of the participants described these symptoms as being due to a mental disorder and proposed different causes such as trauma, personality, or stress. Furthermore, the aggression/harm dimension was less frequently identified as OCD, and the sexual OC content dimension was the least frequently identified as a mental disorder since participants attributed these symptoms to other variables, mainly trauma. Although, in general, participants would recommend psychological treatment, they would more frequently recommend medication for the aggression/harm dimensions and relaxation/meditation for the reactive symptoms. This could suggest that they consider these symptoms to be “less” severe. These results suggest that future intervention programs should describe OCD as a heterogeneous and highly interfering mental disorder that needs empirically validated treatments, describing these treatments, and the ways to access them. As previously emphasized, aggression/harm contents should be carefully described, but these results suggest that content dimensions should be carefully addressed, such as the sexual, doubts/checking, and superstition/symmetry ones.

Some limitations and considerations for future research should be mentioned. This study is part of a larger one Chaves et al. (2022) and the description of the study and consent form included information categorizes it in stigma, mental disorders, and OCD. This information could have biased the results in different ways: participants could be interested in mental health issues and in OCD in particular, and participants could have guessed that the vignette described a person with OCD. To overcome these difficulties and to better describe the attitudes and knowledge of the general population, exclusion criteria included people with some knowledge of mental disorders, as well as having contact with a person with OCD. However, this decision could have biased results in the opposite direction, having a sample not fully representative of the community, with a lower understanding of the nature of OCD, which could result in higher stigma and being less likely to seek help, as previously suggested (e.g., García‐Soriano et al. 2014). Furthermore, we decided to use vignettes in which the gender was not explicit, which seemed interesting to us to help the participants to identify with the person described in the vignette. However, we cannot draw sex‐specific (male/female) conclusions as has been done in other studies that have used vignettes only describing males (Coles et al. 2023; McCarty et al. 2017; Ponzini et al. 2024) or females (Durna, Yorulmaz, and Aktaç 2019). Future studies could explore the differences in the variables of interest depending on sex and taking also into account the sex of the responder. In the same way, to have a “neutral” vignette, we did not describe the race or other identities of the person described in it. It would be interesting to take those varibles into account in future studies to have a more precise picture. Furthermore, although we have used two measures of stigma widely used in the literature such as the AQ and the SDS; they do not include stigmatizing aspects such as perceptions of blame, symptom trivialization, and being a nuisance, which have been seen to be relevant in relation to at least some OC contents in a previous study (Ponzini et al. 2024). Finally, although there is not a general agreement in the literature about whether social desirability has a role in attitudes toward mental disorders (e.g., Griffiths et al. 2006; Norman et al. 2008), it would be interesting for future studies to control this possible influence on participants' responses.

## 5 Conclusions

When the Spanish mental‐health naïve population read vignettes describing people with OCD, that is, people experiencing symptoms associated with significant disturbance and interference in more than one area of their lives, they report stereotypes, feel prejudices, and show an intention to discriminate them. Furthermore, they are only moderately willing to ask for help if any of them experience these difficulties. This is a serious problem that should be tackled in society. Stigma has severe consequences on people's lives and it can be worse than the impact of the mental health condition itself. It is also important to note that although the scores on public stigma were not very high, we should be cautious, as all forms of stigma and discrimination against people with mental disorders, even low or moderate, should be eradicated (Thornicroft et al. 2022). The results show that, as seen in community samples from different American countries, the public stigma and mental health literacy of Spanish mental‐health naïve participants differ among the different OC content dimensions. No differences were found in help‐seeking intention, that is, it is moderately independent of the OCD content.

Delay in seeking treatment is a relevant problem associated with OCD around the world, although differences in this delay have been seen in different populations (García‐Soriano et al. 2014). This study explores the main variables associated with this delay in the Spanish context, with great care taken to ensure OCD content heterogeneity. There was a previous study on Spanish adolescents that only took into account two OC dimensions (García‐Soriano and Roncero 2017). The results are strengthened by the use of six different standardized vignettes describing OCD heterogeneity and by appropriate randomization of each of the vignettes, which allows for cross‐study comparisons of findings. Furthermore, this is the first study integrating the analysis of public stigma dimensions, social distance desire, knowledge about OCD, and help‐seeking intention. Our results support and expand previous studies with an analysis of a European sample and emphasize the need to develop and implement interventions aimed at mitigating help‐seeking barriers and stigma, such as the one recently proposed by Chaves et al. (2022). In this sense, results suggest that programs should describe OCD as a heterogeneous mental disorder, with special emphasis on characterizing autogenous contents as OCD. Based on our results, it will also be necessary to define what a mental disorder is, emphasizing OCD symptoms as well as its significant distress or disability in social, occupational, and other important activities and specifying that all OCD contents are considered an interfering mental disorder that needs professional help. Furthermore, these programs should facilitate the comprehension of OCD. Understanding OCD from a cognitive–behavioral perspective may contribute to a decrease in stigma, such as the perception of people with OCD as being dangerous, which could have an influence on experiencing less anger and fear toward people experiencing OC symptoms and decrease discriminating behaviors (such as avoidance, segregation, or coercion). Finally, describing empirically validated treatments will help users to have less discriminatory behaviors, such as segregation, and increase the probability that they will seek effective treatments.

## Author Contributions

**Antonio Chaves**: conceptualization, data curation, formal analysis, investigation, writing–original draft. **Sandra Arnáez**: data curation, formal analysis, investigation, writing–review and editing. **María Roncero**: data curation, formal analysis, investigation, writing–review and editing. **Gemma García‐Soriano**: conceptualization, funding acquisition, investigation, project administration, supervision, writing–review and editing. All authors reviewed the results and approved the final version of the manuscript.

## Ethics Statement

All procedures described in the study have been approved by the Humans Research Ethics Committee of the Universitat de Valencia, Spain.

## Consent

All study participants provided informed consent prior to study enrolment.

## Conflicts of Interest

The authors declare no conflicts of interest.

### Peer Review

The peer review history for this article is available at https://publons.com/publon/10.1002/brb3.70159.

## Supporting information



Supporting Information

## Data Availability

The data that support the findings of this study are available from the corresponding author upon reasonable request.
