# Supplementary material for: Assessment of Public Stigma, Mental Health Literacy, and Help‐Seeking Intentions Based on Different Dimensions of Obsessive–Compulsive Content: A Study of the Spanish Mental Health‐Naïve Population
Source: Brain Behav. 2024 Dec 15;14(12):e70159. doi: 10.1002/brb3.70159 (PMC11647060; doi:10.1002/brb3.70159)
Supplement: Supplementary file 1 — Supporting Information [file BRB3-14-e70159-s001.docx]

**VIGNETTES**

RELIGIOUS/ BLASPHEMOUS/ IMMORAL

A. is a very religious middle-aged person. He/she often has thoughts of insulting God and cursing other people, he/she thinks that God is going to punish him/her for having such thoughts: “All this makes me very anxious, I come to think that it is better not to go on living if I don’t have God’s approval. Then I lose interest in everything and I have to show God that I respect him and that I don't want my sinful thoughts; I have to pray several prayers in a row, just one is not enough. This brings the situation under control, but even before I finish praying, I have other thoughts again, which forces me to pray other prayers to counteract them.”

These thoughts, together with the time spent praying (several hours a day), cause him/her great discomfort, which hinders his/her family relationships, his/her ability to concentrate and his/her work, which he/she often has to interrupt because of the prayers he/she has to perform.

AGGRESSION/ HARM

A. is a middle-aged person. Thoughts and images of hurting people often come to his/her mind. For example, yesterday, while he/she was having dinner, the thought of attacking his/her partner with the bread knife started to cross his/her mind. As a result, he/she avoids using any sharp objects, and every time he/she has this thought, he/she tries to reason why he/she would not hurt his/her partner. When he/she takes the dog for a walk. If he/she sees a stick on the ground, he/she has thoughts of hitting himself/herself on the head with the stick.

These thoughts cause him/her great discomfort because although he/she does not want to attack or be attacked, he/she is afraid of losing control and doing so. In addition, he/she spends several hours a day checking that he/she is not going to do any harm. All this makes it difficult for him/her at a family level, his/her partner does not understand why he/she refuses to cook with what he/she likes, and why he/she never wants to walk his/her dog anymore. It is also affecting his/her work performance.

SEXUAL

A. is a middle-aged person. Images of unpleasant sexual content often come to his/her mind with people he/she doesn’t want or even disgust him/her. But the ones that upset him/her the most are the images of him/her having oral sex with his/her father. When he/she is with him and the images assault him/her, he/she avoids looking at him and tries to distract himself/herself from thinking (TV or a magazine). For fear of coming into sexual contact with his/her father, he/she avoids any physical contact with him and does not go to visit him as much as he/she would like to. He/she also tries to reason inwardly and convince himself/herself that “it’s OK, I’m not like that...”

These thoughts, and the things he/she does to try to make himself/herself feel better, take up several hours a day and generate great discomfort: *“I can spend more than 2 hours trying to convince myself that I am not going to have oral sex with my father.”* It also hinders his/her performance at work and everyday tasks such as visiting his/her family, making him/her feel like a bad person.

DOUBTS /CHECKING

A. is a middle-aged person. He/she often doubts whether he/she has turned off the gas properly and fears that his/her building will catch fire if he/she leaves it on, which leads him/her to check seven times in a row that it has been turned off. The same thing happens with the taps, both at home and at other people’s houses, he/she turns them off seven times and then checks seven times that no water comes out. He/she is afraid that they are dripping and that there could be a flood. When he/she leaves the house he/she doubts whether he/she has closed the front door and the letterbox properly. He/she has to go back home repeatedly to check seven times that each door is properly closed. He/she does not check more times because he/she is *“*embarrassed in case the neighbors see me*”.*

All this takes up several hours a day and he/she has the feeling that he/she spends all day trying to prevent disasters from happening, something that causes him/her great discomfort, as well as difficulties at family level—his/her partner does not understand his/her repeated checks—and at work since his/her performance has decreased.

SUPERSTITION/ SYMMETRY/ ORDER

A. is a middle-aged person. He/she often feels the need for things to be tidy in a certain way. If they are not, he/she feels great discomfort, and fears that something bad might happen to his/her family. He/she must always carry his/her wallet and mobile phone in the same pocket, something he/she checks repeatedly because if they are not in the same pocket, his family could have an accident. At work, it is essential that everything is tidy in his office, objects are completely aligned, the computer to the left of the papers, and the window completely open. In the kitchen at home and where they eat at work, cutlery, glasses and plates have to be lined up by size with no space between them.

He/she spends several hours a day keeping everything tidy in a certain way for fear that if this is not the case, something that makes him/her live in a state of constant tension that hinders his/her family relationships, and has diminished his/her work performance.

CONTAMINATION/ WASHING

A. is a middle-aged person who works in agriculture and thoughts often come into his/her head about the possibility of being contaminated by using pesticides, insecticides or fertilizers, and of being able to pass the contamination on to other people. He/she thinks that if this happens, it will be his/her responsibility, so he/she feels very uneasy and avoids entering crop fields that have been sprayed or fertilized; using pesticides, insecticides and fertilizers; and touching any agricultural products (fruit or vegetables) that he/she has not been able to wash seven times beforehand. When he/she cannot avoid it and he/she touches something from the field, he/she washes his/her hands repeatedly, keeping the soap on his/her hands until he/she counts to 200. When he/she gets home, he/she takes a shower that lasts approximately 60 minutes.

A. spends several hours a day washing and he/she is finding it difficult to go to the fields every day to look after his/her crops. Since his/her main source of income is farming, these fears are causing him/her economic and family difficulties.
